# Supplementary material for: Tumor Tissue MIR92a and Plasma MIRs21 and 29a as Predictive Biomarkers Associated with Clinicopathological Features and Surgical Resection in a Prospective Study on Colorectal Cancer Patients
Source: J Clin Med. 2020 Aug 4;9(8):2509. doi: 10.3390/jcm9082509 (PMC7465950; doi:10.3390/jcm9082509)
Supplement: Supplementary file 1 [file jcm-09-02509-s001.pdf]

Supplementary Materials: Tumor Tissue MIR92a and Plasma MIRs21 and 29a as Predictive Biomarkers Associated with Clinicopathological Features and Surgical Resection in a Prospective Study on Colorectal Cancer Patients

Masahiro Fukada, Nobuhisa Matsuhashi, Takao Takahashi, Nobuhiko Sugito, Kazuki Heishima, Yukihiro Akao and Kazuhiro Yoshida

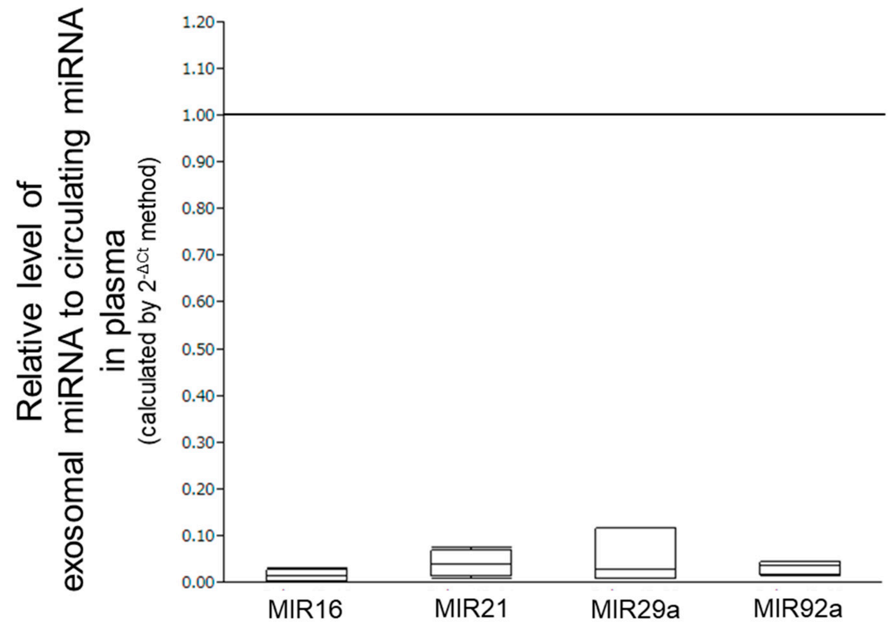

Figure S1. Relative levels of exosomal miRNAs to circulating miRNAs in plasma (MIRs 16, 21, 29a, and 92a).

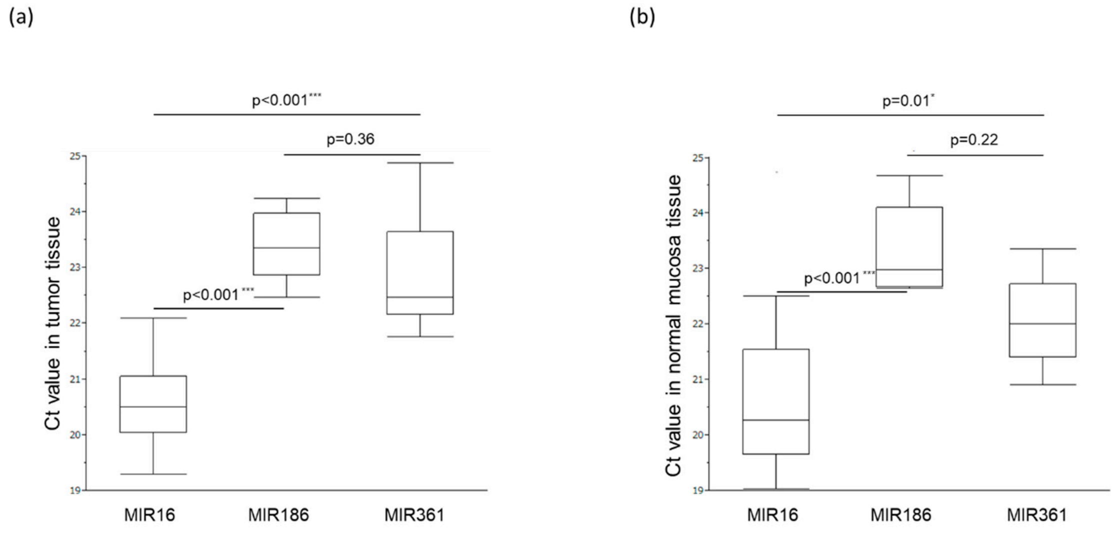

Figure S2. (**a**) Levels of MIRs16, 186, and 361 in tumor tissue from 12 CRC patients. (**b**) Levels of MIRs16, 186, and 361 in normal mucosa tissue of 12 CRC patients. ANOVA followed by Turkey Kramer test was performed for comparisons between groups of MIRs tested.

Table S1. Levels of MIR16, 186, and 361 in colorectal tumor and normal mucosa tissues.

	Target MicroRNAs					
	MIR16		MIR186		MIR361	
	Ct value	*p*-value	Ct value	*p*-value	Ct value	*p*-value
Tumor, median (range)	20.49 (19.29–22.42)	0.76	23.55 (22.47–25.11)	0.86	22.54 (21.76–27.00)	0.23
Normal mucosa, median (range)	20.32 (19.02–25.20)		23.10 (22.64–26.84)		22.04 (20.91–29.81)	

Ct value: Threshold cycle value.

Table S2. Levels of MIR21, 29a, and 92a in colorectal tumor and normal mucosa tissues.

	Target MicroRNAs					
	MIR21		MIR29a		MIR92a	
	−ΔCt value‡	*p*-value	−ΔCt value‡	*p*-value	−ΔCt value‡	*p*-value
Tumor, median (range)	2.58 (1.51–5.91)	<0.001***	1.24 (−0.44–5.75)	<0.001***	1.03 (−0.82–3.87)	<0.001***
Normal mucosa, median (range)	1.30 (0.11–5.07)		0.60 (−0.91–5.68)		0.00 (−0.67–3.62)	

‡: −ΔCt value= −(Ct value (target MIR)–Ct value (internal control MIR16)), ***: $p < 0.001$.
